# Oxide-supported Ir nanodendrites with high activity and durability for the oxygen evolution reaction in acid PEM water electrolyzers†
†Electronic supplementary information (ESI) available. See DOI: 10.1039/c5sc00518c



**DOI:** 10.1039/c5sc00518c

**Published:** 2015-03-27

**Authors:** Hyung-Suk Oh, Hong Nhan Nong, Tobias Reier, Manuel Gliech, Peter Strasser

**Affiliations:** a The Electrochemical Energy, Catalysis, and Materials Science Laboratory , Department of Chemistry , Chemical Engineering Division , Technical University Berlin , Berlin 10623 , Germany . Email: pstrasser@tu-berlin.de

## Abstract

Reducing the noble-metal catalyst content of acid Polymer Electrolyte Membrane (PEM) water electrolyzers without compromising catalytic activity and stability is a goal of fundamental scientific interest and substantial technical importance for cost-effective hydrogen-based energy storage. This study presents nanostructured iridium nanodendrites (Ir-ND) supported on antimony doped tin oxide (ATO) as efficient and stable water splitting catalysts for PEM electrolyzers. The active Ir-ND structures exhibited superior structural and morphological properties, such as particle size and surface area compared to commercial state-of-art Ir catalysts. Supported on tailored corrosion-stable conductive oxides, the Ir-ND catalysts exhibited a more than 2-fold larger kinetic water splitting activity compared with supported Ir nanoparticles, and a more than 8-fold larger catalytic activity than commercial Ir blacks. In single-cell PEM electrolyzer tests, the Ir-ND/ATO outperformed commercial Ir catalysts more than 2-fold at technological current densities of 1.5 A cm^–2^ at a mere 1.80 V cell voltage, while showing excellent durability under constant current conditions. We conclude that Ir-ND/ATO catalysts have the potential to substantially reduce the required noble metal loading, while maintaining their catalytic performance, both in idealized three-electrode set ups and in the real electrolyzer device environments.

## Abstract 2

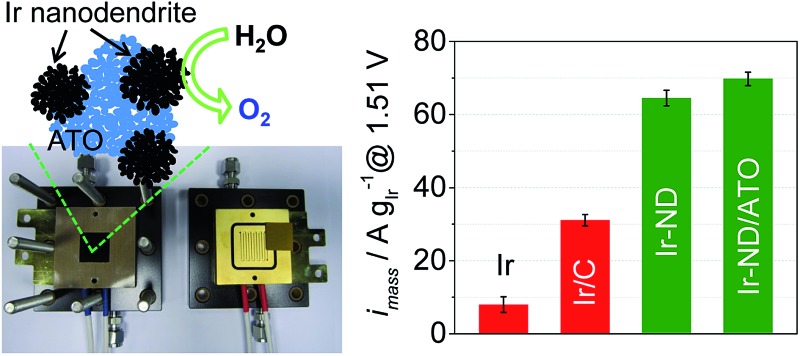
Ir nanodendrites (Ir-ND) supported on antimony doped tin oxide (ATO) show enhanced catalytic activity and stability for oxygen evolution reaction (OER) in polymer electrolyte membrane (PEM) water electrolysis.

## 1. Introduction

Hydrogen is a clean and efficient energy carrier, which can be directly used as a fuel in transportation and various applications.^1,2^ Water electrolysis is one of the most practical ways to produce pure hydrogen, and is well-suited in conjunction with various renewable energy sources, such as wind, solar, geothermal and hydroelectric power.^3–6^ Although alkaline electrolysis is currently used for this purpose, the polymer electrolyte membrane water electrolyzer (PEMWE) technology has received much attention as a promising new alternative technology.^7,8^ Compared to the conventional alkaline electrolysis, PEMWEs show a number of clear advantages, such as high operating current density, energy efficiency, easy operation, compactness and greater operating safety.^9–11^ However, a key limitation of PEMWE is the high cost and high loading of the anodic electrocatalyst for the oxygen evolution reaction (OER). This catalyst is generally incorporates metal oxides of very scarce and expensive precious metal, such as IrO_2_ and RuO_2_.^12–15^ This is why, as the number of the produced PEMWEs units increases, it will become critically important to significantly reduce the amount of these precious metals per PEMWE unit.

One method of achieving this is to maximize the catalytic activity toward OER by designing tailored oxide catalyst shapes with optimized surface-to-mass ratios, thereby minimizing their mass, while maximizing their usage in the PEMWE anode.^16–20^ Among various shape-controlled structures, nanodendrites (ND) have attracted great interest, because the rich edges and corner atoms resulting from a dendritic structure are conducive for high catalytic activity.^21–29^ For instance, Xia *et al.* synthesized Pt–Pd-ND using the seed-mediated method.^26^ The surface of pre-formed Pd seeds provides large number of nucleation sites for the overgrowth of Pt nanoparticles, which was 2.5-fold higher than commercial Pt/C catalysts and 5-fold more active than Pt-black catalysts for oxygen reduction reaction (ORR). Kim and co-workers described the synthesis of Ir-ND by the direct surfactant-mediated method, which showed an extremely high OER activity that was about 5.5 times more active than commercial Ir black.^28^ These results suggest that engineering noble metals into dendritic structure is an attractive approach for achieving high catalytic performance. However, there has been a little study reported on Ir-ND structure for oxygen evolution reaction (OER), which was also restricted to the idealized 3-electrode condition.

Another approach to reduce the amount of noble metal oxides is the use of a high surface area support material, which stabilizes the initial catalyst surface area, and hence the mass based activity of the catalysts.^30–32^ Typically, carbon black has been used as catalyst support material.^33–35^ However, carbon material is easily corroded in the strongly oxidative condition of water electrolyzers, and such electrochemical corrosion leads to the aggregation and migration of noble metal catalysts and even their detachment from the support surface with a loss of the electroactive surface area.^36–38^ Therefore, several conductive metal oxides including TiO_2_, Nb_2_O_5_, SnO_2_ and others have been investigated as an alternative to carbon.^39–44^ Among these, antimony doped tin oxide (ATO) with relatively high electronic conductivity has been proposed as a good candidate as support material.^45–49^ Scott *et al.* synthesized ATO supported IrO_2_ catalysts for OER, which showed some improved activity compared to the pristine IrO_2_ in single cell studies.^48^ The authors also demonstrated that the higher performance of the supported catalysts was mainly attributed to better dispersion of IrO_2_ on ATO compare to pristine IrO_2_. Wang and co-worker also used the composite materials consisting of ATO and Cs-substituted phosphor-tungstate as the support of iridium oxide.^49^ The mass-specific catalytic activity of the synthesized IrO_2_/Cs_1.5_HPA–ATO material was higher compared to IrO_2_. However, the relatively low BET surface area of ATO severely limited the mass catalytic activity and stability. Similar conclusions were proposed by other groups.^45,46^ In our previous work, we successfully synthesized the tin doped metal oxides (ATO, FTO, ITO) with a specific surface area of 125–263 m^2^ g^–1^, which showed the higher corrosion resistance than that of the carbon black.^50^

In the present study, we prepare and characterize Ir-nanodendrite (Ir-ND) OER catalysts and demonstrate their exceptionally high activity and stability during the OER catalysis. Ir-ND were synthesized and supported on high surface-area mesoporous antimony-doped tin oxide (ATO). The electrocatalytic activity and stability of Ir-ND/ATO were investigated using cyclic voltammetry and galvanostatic chronopotentiometric experiments. The physical properties were tested *via* XRD, BET and HR-TEM. Beyond the idealized 3-electrode tests, catalytic activity and stability tests in real single cell PEM electrolyzer were conducted and compared. Our study evidences that Ir-dendrites supported on ATO constitute a novel, effective catalyst concept with significant proven performance benefits for PEM water electrolyzers.

## 2. Experimental

### 2.1 Synthesis of ATO support

Antimony doped tin oxide (ATO) with high surface area was synthesized using a combined sol–gel and hydrothermal method described in our previous work. 1.28 g of tetradecylamine (TDA, CH_3_(CH_2_)_13_NH_2_, 95%, Sigma Aldrich) was dissolved in ethanol solution (EtOH 65 mL, D.I. water 160 mL) and the stirring was continued for 3 hour. 4.794 g of tin tetrachloride (SnCl_4_, 99.995%, Sigma Aldrich) and 0.48 g of antimony(iii) acetate ((CH_3_CO_2_)_3_Sb, 99.99%, Sigma Aldrich) were dissolved in 20 mL of ethanol followed by adding into the TDA solution. After stirring for 1 hour, the mixture was added to 200 mL ammonium hydroxide solution (1.5 mmol L^–1^). The resulting suspension was refluxed for 72 hour at 80 °C and then cooled down to room temperature. The white precipitate was separated with solution by centrifugation (8200 rpm, 15 min), and then washing was repeated 5 times with water. The as-prepared wet sample was transferred to a glass-lined stainless-steel autoclave and hydrothermally treated at 120 °C for 24 hour. To remove the excess surfactant, final product was centrifuged and washed with ethanol–water (volume ratio 1 : 1) for 5 times. Produced bright yellow powder was dried in a freeze dryer and then calcined at 400 °C for 3 hour in air condition.

### 2.2 Preparation of the Ir nanodendrites (Ir-ND)

For the synthesis of the Ir nanodendrites (Ir-ND), 0.01 mmol dihydrogen hexachloroiridate(iv) hydrate (H_2_IrCl_6_·*x*H_2_O, 99%, Alfa Aesar) and 1 mmol tetradecyltrimethylammonium bromide (TTAB, ≥99%, Sigma Aldrich) were dissolved in 7.5 mL de-ionized water. 5 mg of sodium hydroxide (NaOH, 97%, Sigma Aldrich) was placed in the mixture, followed by heating to 70 °C with vigorous stirring for 30 min. Then, ice-cold NaBH_4_ solution (150 mM, 2.5 mL) was rapidly added to the mixture under stirring and the reaction mixture was maintained at 70 °C for 6 hour. The resultant solution was cooled down to room temperature naturally. The resulting colloidal products were collected by centrifugation and washed three times with ethanol–water mixtures (volume ratio 1 : 1). Ir-ND nanoparticles were dispersed into an ethanol solution and the appropriate amount of synthesized ATO was added to the solution under vigorous stirring. Subsequently, Ir-ND deposited on ATO with ultrasonic dispersion for 1 hour and then stirred at room temperature for 24 hour. After that, the Ir-ND/ATO was filtered and dried in a freeze dryer.

### 2.3 Electrochemical characterization and MEA test

Electrochemical characterization of Ir-ND was performed using a rotating disk electrode (RDE, Pine Research instrument). The experiment was performed in a three-electrode electrochemical cell in 0.05 M H_2_SO_4_. A glassy carbon electrode (0.196 cm^2^) with a thin film of the prepared sample was used as the working electrode. A platinum wire and mercury/mercurous sulfate (MMS, Hg/Hg_2_SO_4_) electrode were used as the counter and reference electrodes, respectively. Prior to OER measurement, the surface of the synthesized Ir-ND was oxidized using a potential sweep between 0.05 and 1.5 V_RHE_, 50 cycles at a scan rate of 500 mV s^–1^. In this step, TTAB on the surface of Ir-ND was successfully removed.^51^ The electrochemical porosity, activity and stability of synthesized electrocatalysts were analyzed, and details about electrochemical protocols were provided in the ESI.†

Membrane electrode assemblies (MEA) with the synthesized catalysts as anode were prepared using Nafion 212 membrane by the catalyst coated membrane (CCM) method. A commercial Pt/C (46 wt%, TKK) was used as the cathode. Catalyst inks were prepared by ultrasonically mixing 5 wt% Nafion ionomer in isopropanol (IPA), and then they were sprayed directly onto the membrane with a 5 cm^2^ geometric area. The polarization curves were measured at 80 °C and atmospheric pressure. D.I. water was pumped using a peristaltic pump to the anode and cathode side, which was pre-heated to the 80 °C by heating circulator. The detail experiment was described in the ESI.†

### 2.4 Physical characterization

High-resolution transmission electron microscopy (HR-TEM) was carried out by using FEI Tecnai G2 20 S-TWIN operated at 200 kV, which was performed to observe structure and particle size of synthesized electrocatalysts. EDX was employed to study the element composition of Ir supported on ATO. X-ray diffraction (XRD, Bruker D8 Advance instrument, Cu Kα radiation) was used to examine the crystal structure and identify the nature of electrocatalysts. The surface area was determined by nitrogen adsorption and desorption measurements (Quantachrome Autosorb-1-C). Brunauer Emmett Teller model (BET) formulations were used to calculate the surface area and non-local density functional theory (NLDFT) model was employed to analyze the pore size distribution of Ir-ND.

## 3. Results and discussion

### 3.1 Morphology, structure, and porosity of Ir nanodendrite (Ir-ND) electrocatalysts

**Fig. 1:**
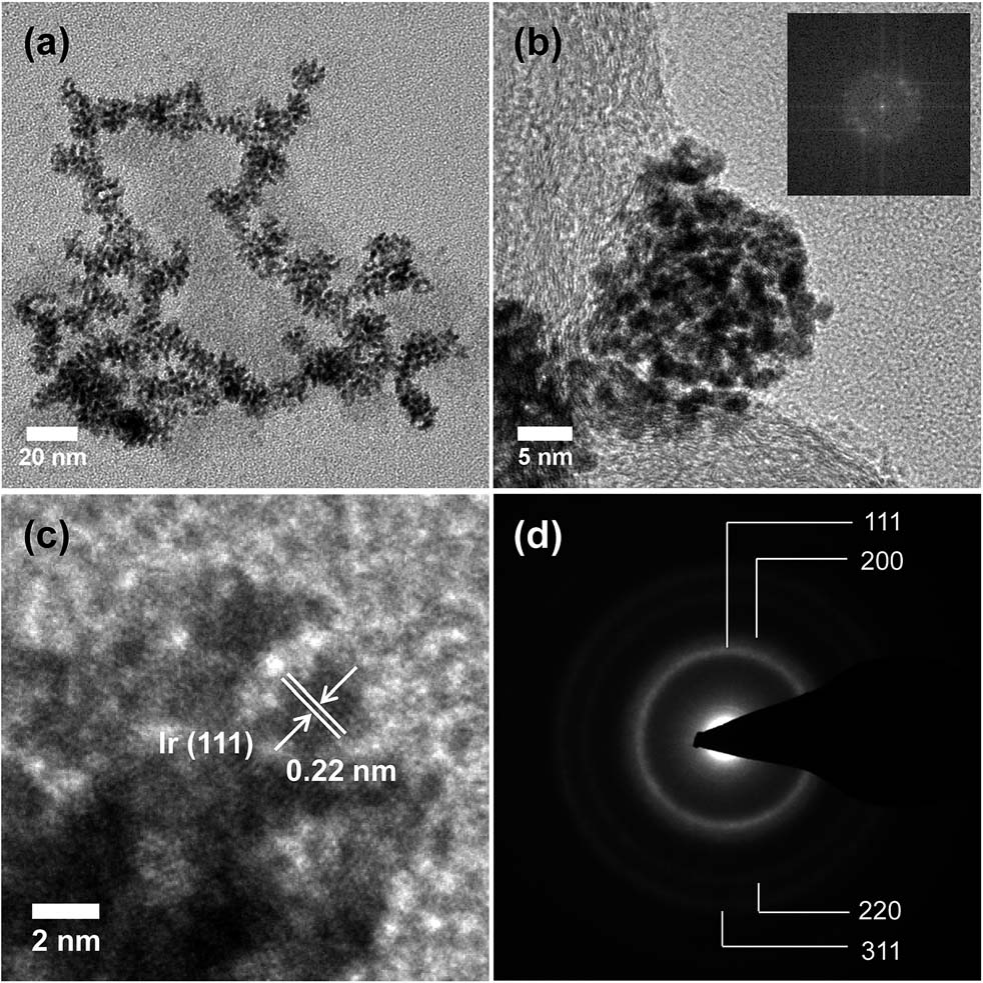
(a) Low and (b) high resolution TEM images of the Ir nanodendrites (Ir-ND) synthesized by reducing H_2_IrCl_6_·*x*H_2_O with NaBH_4_ in the presence of TTAB and the corresponding Fourier-transform (FT) pattern of the Ir-ND (inset b). (c) Magnified HRTEM image of one single Ir-ND and (d) the corresponding SAED pattern.

Ir-ND synthesized by the direct surfactant-mediated method, which is based on the self-assembly of tiny metal seeds. On the basis of this mechanism, Ir is nucleated in a short time due to the strong reducing agent, which leads to the rapid formation of large number of tiny Ir-seeds. These small Ir-seeds are self-assembled to reduce the huge surface energy and TTAB was used to guide the attachment of Ir-seeds into dendritic structure.^52^ Fig. 1 shows representative TEM images of the branched Ir-ND. The low resolution TEM image of the as-synthesized material revealed a largely dendritic structure with a narrow size range of 15–20 nm (Fig. 1a) and high-yield formation. Fig. 1b depicts a higher resolution TEM (HR-TEM) image, indicating that the single Ir-ND possessed porous nanostructures with multi-branched subunits, and consists of short-rod shaped nanoparticles. The corresponding Fourier-transform (FT) of the Ir-ND image in Fig. 1b showed sharp distinct diffraction maxima, evidencing the single crystalline structure rather than an agglomeration of the nanoparticles.

Higher magnification images revealed a lattice spacing of 0.22 nm (Fig. 1c), which was close to the Ir (111) plane of the face-centered cubic (fcc). Fig. 1d shows a selected-area electron diffraction (SAED) pattern of a single Ir-ND with its very bright concentric rings attributed to (111), (200), (220) and (311). This result suggested that the Ir-NDs were poly-crystalline in nature with enhanced (111) diffraction. The composition of Ir-ND was further investigated by TEM-based energy dispersive X-ray spectroscopic (EDX) analysis, which showed a contamination-free pure Ir composition (see Fig. S1 in ESI†).

**Fig. 2:**
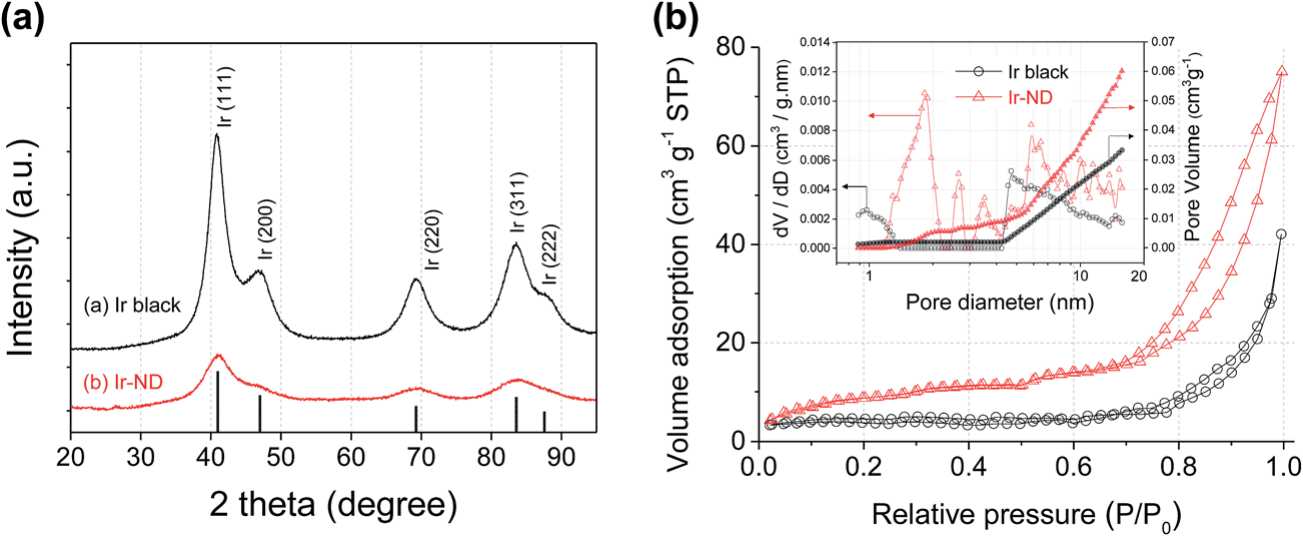
(a) XRD patterns of commercial Ir black and synthesized Ir-ND. (b) Nitrogen adsorption–desorption isotherms and pore size distribution (inset) of commercial Ir black and synthesized Ir-ND. Pore size distribution was calculated using non-local density functional theory (NLDFT).

Powder diffraction (XRD) (Fig. 2a) was performed to compare the long-range order and crystal structure of the Ir-ND to commercial Ir black (Alfa Aesar, 99.8%). Five major diffraction peaks at 40.6, 47.3, 69.1, 83.4 and 88.2° were indexed to the (111), (200), (220), (311) and (222) reflections of the fcc Ir metal (JCPDS Card no. 87-0715), respectively. The crystallite size of Ir-ND and Ir black was estimated to 1.7 nm and 3.2 nm, respectively. Further, the pore structure of the Ir-ND and Ir black were measured using nitrogen adsorption–desorption isotherms (Fig. 2b): the isotherm curve of Ir-ND showed a typical IV curve with a response characteristic of porous materials.^53,54^ Furthermore, the hysteresis loop resembled a H3 type, which is usually associated with complex interconnection of slit shaped pores.^55,56^ This isotherm result demonstrated that there was large number of pores in the Ir-ND, agreeing well with earlier TEM images (see Fig. 1). In contrast, Ir black showed a type III isotherm without the pore structure and this result was also confirmed with the observation from TEM studies (see Fig. S2 in ESI†).^53,54^ As shown in the inset of Fig. 2b, pore size distribution was calculated using the non-local density functional theory (NLDFT) model.^57,58^ Ir-ND exhibited rather small pore diameter (1.9 nm) and high pore volume (0.072 mL g^–1^) as compare with Ir black (4.6 nm, 0.052 mL g^–1^). The calculated BET surface area of Ir-ND and Ir black were 39.2 m^2^ g^–1^ and 18.2 m^2^ g^–1^, respectively.

### 3.2 *In situ* surface redox chemistry of Ir-ND electrocatalysts

**Fig. 3:**
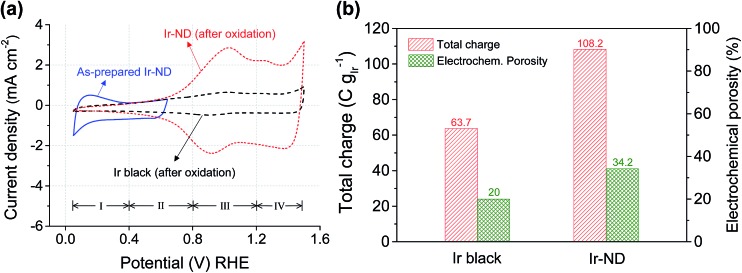
Surface redox electrochemistry of commercial iridium black and iridium nanodendrites (Ir-ND): (a) cyclic voltammograms (CVs) of as-prepared and oxidized nanodendrites. Conditions: 25 C, N_2_-purged 0.05 M H_2_SO_4_, 20 mV s^–1^. The CV of the as-prepared Ir nanodendrites (Ir-ND) was recorded between +0.05 to +0.64 V_RHE_ to avoid Ir oxide formation. (b) Electrochemical total charge and porosity of Ir-ND and Ir black.

To evaluate the surface electrochemical properties of the prepared Ir-ND catalysts, cyclic voltammograms (CV) were measured in 0.05 M H_2_SO_4_ solution with a sweep rate of 20 mV s^–1^ (see Fig. 3a). In general, the solid-state redox-transitions of metal oxides are accompanied by ion exchange processes reflected by characteristic redox waves across the considered potential window.^59,60^ In particular for Ir oxides, the redox processes can be concisely represented by the equation

1 IrO_*x*_(OH)_*y*_ + *δ*H_(solution)_^+^ + *δ*e_(oxide)_^–^ → IrO_*x*–*δ*_(OH)_*y*+*δ*_

The CV of Ir-ND in Fig. 3a also showed the characteristic charging/discharging peaks of the redox pseudo-capacitance and double-layer capacitance. When repetitive potential cycling was applied for the fresh Ir-ND catalyst between +0.05 V_RHE_ and +0.64 V_RHE_ to avoid excessive bulk oxide formation, underpotential hydrogen adsorption–desorption peaks (region I) appeared similar to other noble metals like Pt.^61^ However, after oxidation of the Ir surface through potential cycles between +0.05 and +1.5 V_RHE_ the characteristic H_upd_ peaks of Ir-ND and Ir black disappeared completely, while two new redox peaks emerged indicating the formation of higher Ir-oxide species (see regions III and IV in Fig. 3a). The two redox waves can be associated with the Ir(iii)/Ir(iv) and Ir(iv)/Ir(>iv) redox couples, respectively.^62^ In particular, Ir(0) is first oxidized to Ir(iii) at low potentials (region II) and subsequently to a hydrous Ir(iv) oxide species in potential region III. Further anodic above +1.2 V (region IV), the redox features involve the oxidation of Ir(iv) to a higher valence, most likely to Ir(vi) and Ir(v).^63^ Based on above reaction mechanism, we concluded that both catalysts were irreversibly converted to a hydrous Ir oxide (IrO_*x*_) with the concurrent loss of their metallic Ir character.

*In situ* measurement of the active surface area and pore structure of electrocatalysts under catalytic conditions were achieved by looking at the electrochemical outer (q*o), inner (q*i), and total (q*T) charge (Fig. 3b and S3†).^64,65^ The calculated electrochemical total charge and porosity of Ir-ND were 108.2 C g_Ir_^–1^ and 34.2%, which were 1.7 times higher than those of Ir black (63.7 C g_Ir_^–1^, 20%), respectively. This is in excellent agreement with the BET results and corroborates the earlier conclusions of the TEM morphology analysis.

**Fig. 4:**
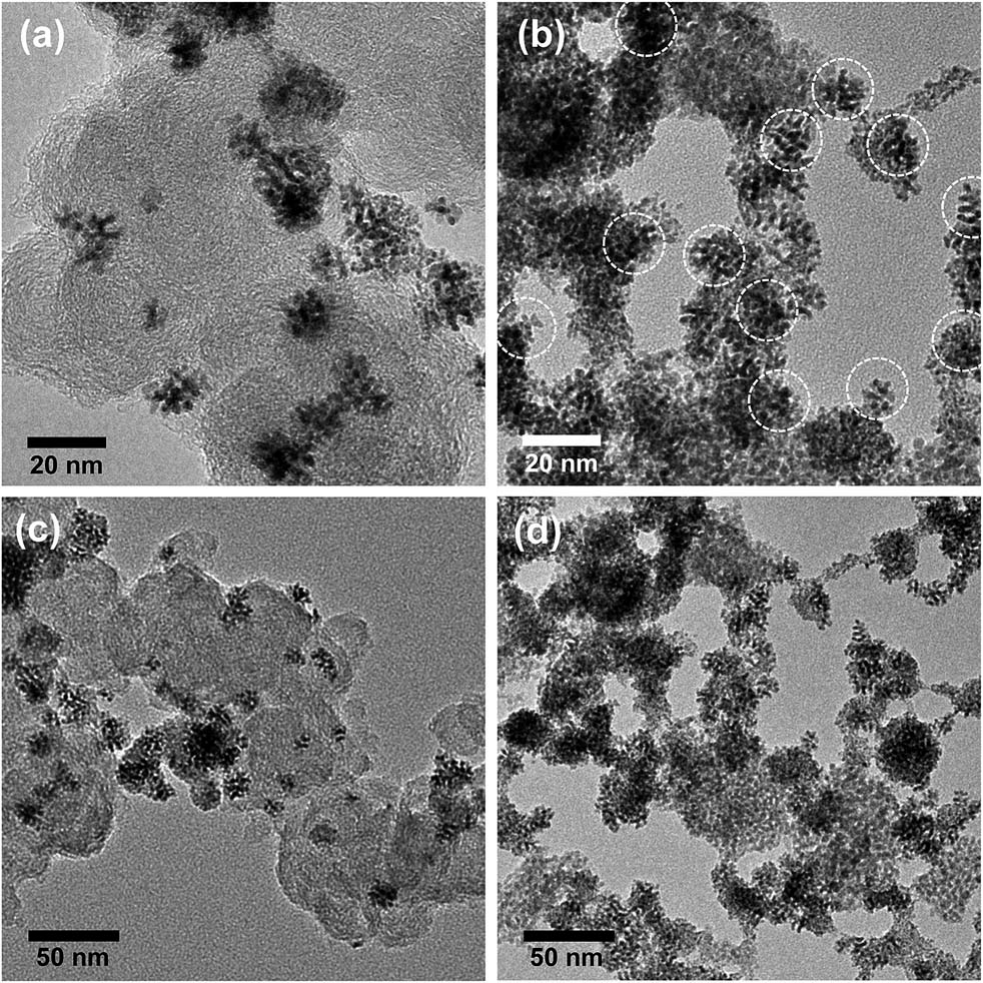
HR-TEM images of Ir-ND supported on (a) carbon black and (b) antimony-doped tin oxide (ATO). Low magnification TEM images of (c) Ir-ND/C and (d) Ir-ND/ATO.

To achieve an additional reduction in noble metal loadings, the Ir-ND catalysts were supported on both high-surface area carbon blacks and high-surface area Sb-doped tin oxides (ATO) (Fig. S4†), denoted as Ir-ND/C and Ir-ND/ATO, respectively. Both support materials exhibited large surface area and analogous pore volume of 263 m^2^ g^–1^, 0.408 mL g^–1^ for ATO compared to 235 m^2^ g^–1^, 0.554 mL g^–1^ for carbon black. A detailed morphological analysis of the supported catalysts (Fig. 4) evidenced an even distribution of the nanodendrites (ND) at 15–20 nm. Compositional EDX analysis revealed a successful contact anchoring of the Ir-ND on the ATO supports (Fig. 4b and S5†).

### 3.3 Electrocatalytic activity of Ir-ND catalysts for acid water splitting

**Fig. 5:**
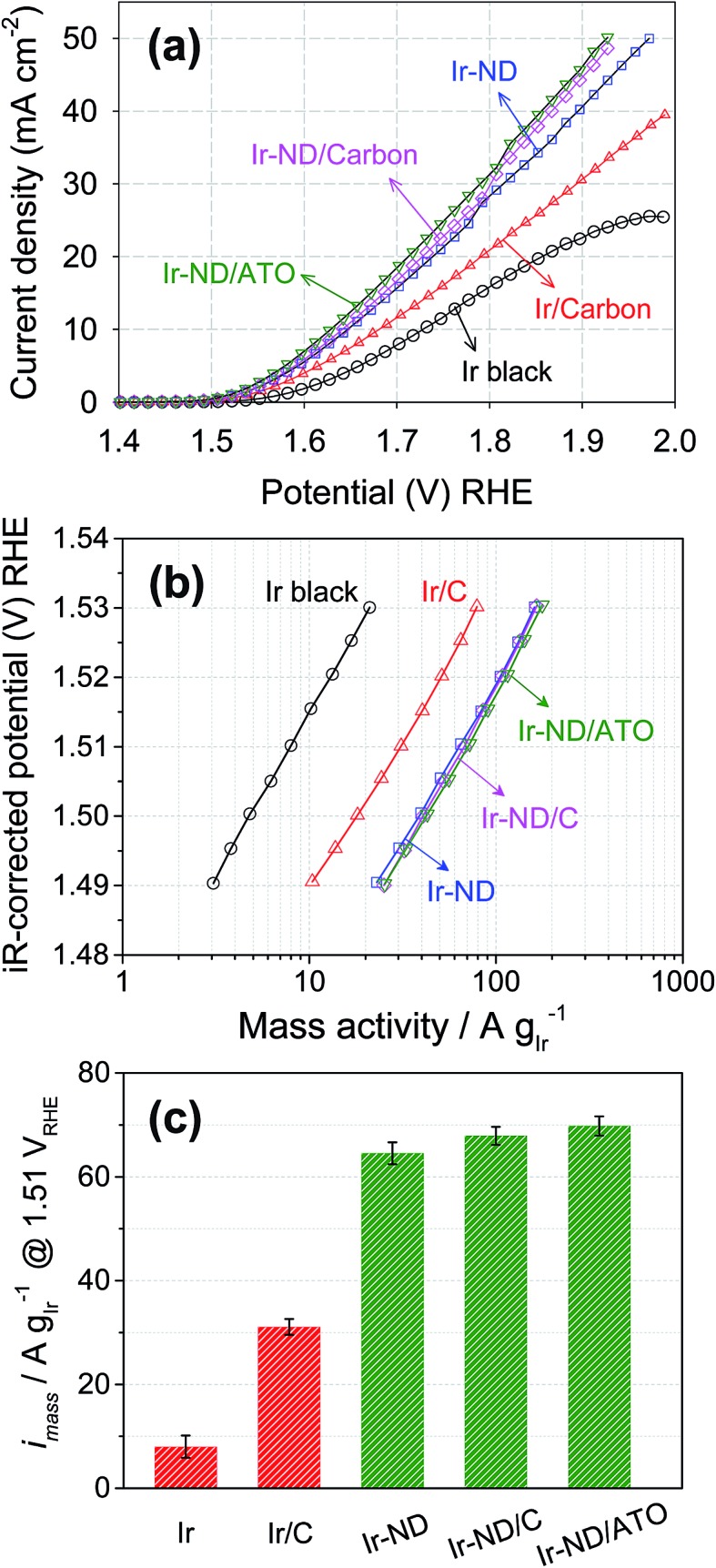
The electrocatalytic OER activities of Ir-ND, Ir-ND/C and Ir-ND/ATO. For comparison, commercial Ir black and synthesized Ir/C by polyol process were also evaluated under same condition. (a) The polarization curve for OER of electrocatalysts in three electrode system, (b) *iR*-corrected Tafel plots from the polarization curve, (c) Ir-mass based activity at *η* = 280 mV overpotential (1.51 V_RHE_). All the measurements were performed in N_2_-purged 0.05 M H_2_SO_4_. Catalysts loading: 10.2 μg_Ir_ cm^–2^. Scan rate: 5 mV s^–1^.

The catalytic activity for the oxygen evolution reaction, OER, of the Ir-ND electrocatalysts was examined in acid electrolytes using linear sweep voltammetry. Fig. 5a shows OER polarization curves of unsupported Ir-ND, carbon-supported Ir-ND/C, and oxide-supported Ir-ND/ATO catalysts after an initial electrochemical oxidative break-in treatment. For comparison, Ir black and Ir nanoparticles supported on carbon black (Ir/C, see Fig. S6†) were also investigated under same condition. Clearly, the oxide-supported Ir-ND catalyst significantly outperformed all Ir reference catalysts. The OER catalytic activity rose in the order: Ir black ≪ Ir/C ≪ Ir-ND < Ir-ND/C < Ir-ND/ATO. We attribute the enhanced OER activities of unsupported Ir-ND electrocatalysts over unsupported Ir black to their intrinsic dendritic structure. On top of the intrinsic morphology, further improvement in OER catalytic activity was achieved by the stable dispersion of the Ir-ND on the support surface exposing an increased number of active sites. IR-corrected Tafel plots from the fits of polarization curves are given in Fig. 5b and Table S1.† The obtained Tafel slopes of 55.6–57.7 mV dec^–1^ are close to the theoretical value of 60 mV dec^–1^. Notwithstanding ambiguities about its detailed mechanistic significance in complex multi-electron reactions, Tafel slopes of about 60 mV dec^–1^ have traditionally been associated with a rapid electrochemical equilibrium reaction followed by a non-faradaic, chemical rate-determining reaction step, as reported earlier for IrO_2_ in acidic medium electrolyte:^13,66–70^

2 S + H_2_O ⇔ S–OH*ads + H^+^ + e^–^

3 S–OH*ads ⇒ S–OH_ads_

where S represents a suitable active catalyst site. The adsorption intermediates S–OH_ads_ and S–OH*ads were believed to possess a similar chemical structure, but different energies. An alternative formal interpretation of the 60 mV dec^–1^ Tafel slope would involve an one-electron electrochemical rate-determining step with an anodic charge transfer coefficient *α*_a_ = 1, indicating that the transition state is structurally close to the final state of the rate-determining step. In line with recently proposed trends in oxygen binding of metal oxides and free-energy reaction pathways for the OER, a suitable rate-determining step for Ir oxide is the formation of a peroxide species from an absorbed oxygen atom, O_ads_ according to

4 S–O_ads_ + H_2_O → S–OOH_ads_ + H^+^ + e^–^.

In a subsequent coupled electron/proton charge-transfer step, the peroxide, OOH_ads_, was predicted to yield molecular oxygen, O_2_.^71–75^ Other factors often affecting the kinetic Tafel slope of complex reactions include adsorbate coverage-dependent linear-free energy relationships, *e.g.* following Tempkin isotherms. This brief discussion demonstrates why in absence of detailed atomic-scale experimental evidence reliable mechanistic interpretations of the experimental Tafel slope remain elusive.

In order to assess the more technical precious metal-based catalyst performance, we compared the Ir-mass based activities at an overpotential of *η* = 280 mV (= 1.51 V_RHE_ electrode potential) of all five catalysts. As illustrated in Fig. 5c and Table S1,† Ir-ND electrocatalysts exhibited a twofold higher activity compared to the carbon-supported Ir (Ir/C), but were 8-times more active for OER than the commercial Ir black. These excellent noble-metal mass-based activities of Ir-ND are due to their large surface area, large number of edges and corners atomic steps, all of which often result in much higher reaction activities.^26,76^ The recently proposed metal-mass based activity benchmark level of 10 A mg_Ir_^–1^ was reached at a previously unachieved electrode potential of below 1.48 V.^6^ Being below the thermo-neutral voltage of water splitting, this performance underlines the energy efficiency of the Ir-ND catalysts.

### 3.4 Water splitting performance of Ir-ND in single cell PEM electrolyzers

Three-electrode RDE half-cell tests are an important tool to assess the short-term, small scale performance of a new electrocatalyst. Years of catalyst development in the PEM fuel cell area have shown, however, that RDE ultimately remains a screening tool that often fails to reliably predict activity or stability in a full galvanic or electrolytic device.^77^

**Fig. 6:**
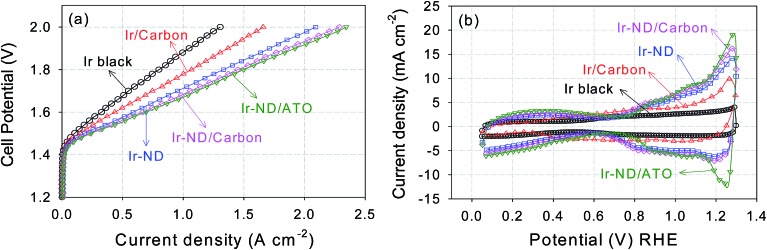
Electrocatalytic performance of Ir-ND catalysts in a single PEM electrolyzer cells: (a) Membrane Electrode Assembly (MEA) polarization curves with a catalyst loading of 1.0 mg cm^–2^ and (b) cyclic voltammograms, 50 mV s^–1^. Other conditions: 80 °C cell temperature, 5 cm^2^ active electrode area, preheated 80 C deionized water was supplied at 1.5 mL min^–1^.

Thus, to assess the performance of our novel Ir-ND catalysts under more realistic conditions, single Membrane Electrode Assemblies (MEAs) were prepared using the oxide-supported Ir-ND as anode, commercial NR-212 membranes, and commercial Pt as cathode electrocatalysts. Their water splitting performance was then evaluated in a realistic single polymer electrolyte membrane water electrolysis cell (Fig. 6a). Again, for comparison, commercial Ir black and carbon black supported Ir nanoparticles were evaluated under same conditions. In line with the half-cell tests of Fig. 5a, the MEA performance of Ir-ND/ATO was superior to all tested materials, exhibiting a *technologically relevant current density* of 1.50 A cm^–2^ at 1.8 V. Water splitting current densities of Ir-ND/C and Ir-ND reached 1.45 and 1.33 A cm^–2^ at 1.8 V, respectively. We explain that based on the improved dispersion of the supported catalyst. Next came the Ir/C catalyst with a current density of 1.07 A cm^–2^ and finally the (technologically employed) unsupported Ir black catalyst (0.80 A cm^–2^). Note that the CV of the novel Ir-ND catalysts showed much higher voltammetric charge at 0.9–1.2 V_RHE_, which is an indication of the electrochemical surface area for OER, as shown in Fig. 6b.^78^ This is attributed to a more pronounced surface redox transition of Ir(iii)/Ir(iv), suggesting a larger number of exposed Ir active sites. In comparison to the open literature, our Ir-ND/ATO showed higher current density, even compared to IrO_2_ using a colloidal method, which permitted a lower heat treatment temperature, its performance was enhanced.^79^ In particular, Ir-ND/ATO (693 A g_Ir_^–1^ cm^–2^) exhibited 2.3 times higher mass-normalized current density than colloidal IrO_2_ (300 A g_Ir_^–1^ cm^–2^) at 1.6 V.

### 3.5 The electrochemical stability of Ir-ND water splitting catalysts

**Fig. 7:**
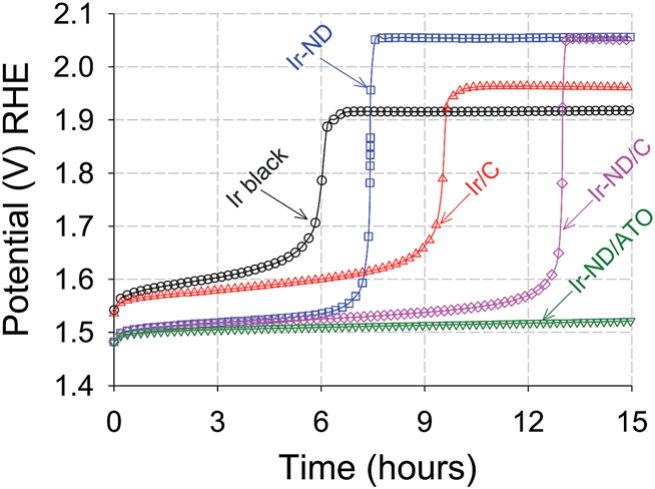
Accelerated constant current-load durability test (ADT) of Ir-ND OER electrocatalysts in comparison to reference catalysts measured under identical conditions. test time: 15 h, N_2_-purged condition, catalyst loading: 10.2 μg_Ir_ cm^–2^, 1600 rpm, 10 mA cm^–2^.

Accelerated durability tests of Ir-ND, Ir/C and Ir black electrocatalysts were conducted at constant current densities of 1 mA cm^–2^ for 15 hours in liquid acid, Fig. 7. All measured electrode potential gradually increased, suggesting a certain degree of voltage instability due to corrosion or particle sintering. Again, our novel Ir-ND catalyst outperformed all other reference catalysts. The overall voltage stability of the electrocatalysts increased in the order: Ir black < Ir-ND < Ir/C < Ir-ND/C < Ir-ND/ATO. The supported electrocatalysts exhibited a clearly more stable behavior (slower potential increase) than the catalyst blacks. The much longer life time of the supported Ir-ND/ATO is primarily attributed to the introduction of the ATO support with high surface area and also possessed high corrosion resistance in acidic medium.^50^

## 4. Conclusion

In conclusion, we have presented a promising novel family of nanostructured Ir water splitting catalysts, referred to as Ir nanodendrites (Ir-ND). The Ir-ND showed all the beneficial physico-chemical attributes required for a element-efficient catalyst, such as a large surface area, small mean particle size, combined with a high electrochemical porosity (34.2%) when compare with commercial Ir blacks. Supported on corrosion stable oxides, the Ir-ND exhibited an unprecedented electrochemical performance for the oxygen evolution reaction (OER) in idealized three electrode set ups. Furthermore and more importantly, the Ir-ND confirmed their RDE-based activity benefit in realistic single cell PEM electrolyzer testing. Ir-ND/ATO by far outperformed Ir blacks and Ir/C. The electrochemical durability of Ir-ND/ATO surpassed that of the reference catalysts. Based on our present data and conclusions, we believe that the development and deployment of nanostructured Ir-ND/ATO catalysts constitute a substantial progress toward low noble-metal OER electrocatalysts for acid PEM electrolyzers.
