# Supplementary material for: Oxide-supported Ir nanodendrites with high activity and durability for the oxygen evolution reaction in acid PEM water electrolyzers
Source: Chem Sci. 2015 Mar 27;6(6):3321–8. doi: 10.1039/c5sc00518c (PMC5490338; doi:10.1039/c5sc00518c)
Supplement: Supplementary file 1 [file SC-006-C5SC00518C-s001.pdf]

Electronic Supplementary Material (ESI) for Chemical Science.
This journal is © The Royal Society of Chemistry 2015

*Supporting Information*

# Oxide-supported Ir nanodendrites with high activity and durability for the oxygen evolution reaction in acid PEM water electrolyzers

Hyung-Suk Oh, Hong Nhan Nong, Tobias Reier, Manuel Gliech and Peter Strasser *

*The Electrochemical Energy, Catalysis, and Materials Science Laboratory, Department of Chemistry, Chemical Engineering Division, Technical University Berlin, Berlin 10623, Germany.*

* Corresponding author:

Tel: 49 30 314 29542

Fax: 49 30 314 22261

E mail: pstrasser@tu-berlin.de

***Experimental***

## ***1. Preparation of Ir/C***

In a synthesis of Ir supported on carbon (Ir/C), 0.2 mmol of $Ir(CH_3COO)_3$ and 80 mg of $NaOH$ were dissolved in 20 mL of ethylene glycol under vigorous stirring for 30 min. 29.5 mg of carbon black (Vulcan) was added to the mixture to produce 20 wt.% of Ir/C. The resulting solution was ultrasonicated for 20 min and then refluxed for 30 min at 160 $^{o}$C under nitrogen condition. The solution was allowed to cool down to room temperature and left for 24 hour with continuous stirring. The reaction solution was centrifuged (8200 rpm, 15 min) and washed several times with ultrapure water. The collected Ir/C slurry was dried in a freeze dryer followed by heat treated at 250 $^{o}$C in nitrogen atmosphere for 2 hour.

## ***2. Physical property of metal oxide:***

***Transmission electron microscopy (TEM):*** The prepared Ir-ND nanoparticles dispersed in ethanol and deionized water (D.I. water) (1:1), and then deposited onto carbon coated copper grids and dried at 50 $^{o}$C in convection oven. These samples were observed under a FEI TECNI G2 20 S-TWIN equipped with LaB6 cathode by an acceleration voltage of 200 kV. An energy dispersive X-ray (EDX) spectroscopy detector and a GATAN MS794 P CCD camera were employed to investigate the Ir-ND particle morphology and overall elemental composition of Ir-ND/ATO and Ir-ND/C.

***Wide-angle X-ray Diffraction (XRD):*** Powder X-ray diffraction patterns (XRD) of the prepared samples were obtained with a D8 Advanced Bruker AXS diffractometer using Cu Kα radiation. Data were collected in a $2\theta$ range of 20° to 95° and the XRD profiles were fitted with the Pearson VII function using the software Jade 8. Particle sizes (D) were

calculated from X-ray diffraction data using the Scherrer formula:

$$Particle\ size\ (D) = {}^{K \cdot \lambda}\!/_{FWHM \cdot cos\theta}$$

where $K$ = Scherrer constant, which is generally taken to have the value 0.9,

$\lambda$ = wavelength of x-rays,

$FWHM$ = full width at half maximum of the reflection peak that has the same maximum intensity in the diffraction pattern,

$\theta$ = diffraction angle of x-rays

***Physisorption:*** The textural property of the prepared Ir-ND was obtained by nitrogen adsorption and desorption measurements at 77 K using a Quatachrome Autosorb-1-C system. For comparison, commercial Ir black was also measured under same condition. Samples were outgassed at 150 $^{o}$C under vacuum line for at least 2 h before each measurement. The surface area was calculated by the Brunauer-Emmett-Teller (BET) method, by using the Quantachrome$^{TM}$ AS1Win$^{TM}$ software. The Branuer-Emmett-Teller (BET) method was utilized to calculate the surface area, according to the bellow equations (E1) – (E2):

$$\frac{{}^{p}/_{p_0}}{n(p_0-p)} = \frac{1}{n_m C} + \frac{C-1}{n_m C}\frac{p}{p_0} \qquad \text{(E1)}$$

$$A_s(BET) = n_m N_A a_m \qquad \text{(E2)}$$

Here, $p$ is the measured pressure, $p_0$ is the saturation pressure of the adsorptive, $C$ is the BET constant, $n_m$ is the monolayer capacity, $n$ is the amount of the adsorbent, $A_s$ is the BET surface area, $N_A$ is the Avogadro constant, $a_m$ is the cross-sectional area of the

adsorptive molecules. The pore-size distribution was also calculated by using the Quantachrome™ AS1Win™ software. The calculation model was the hybride cylindrical/sphere pore, non-local density functional theory (NLDFT) adsorption model. This simulation is achieved by fitting the adsorption isotherm and using a fast non-negative least-square algorithm to solve the generalized adsorption isotherm equation (E3).

$$N(P/P_0) = \int_{r_{min}}^{r_{max}} N\,(P/P_0\,, r) f(r) dr \quad \text{(E3)}$$

This gives the experimental isotherm $N(P/P_0)$ where, $r$ is the pore width, $f(r)$ is the pore-size distribution function, and $N(P/P_0\,, r)$ is the isotherm on a single pore of $r$.

### ***3. Electrochemcial study:***

***Preparation of sample ink:*** The sample ink was prepared by blending the 5.0 mg of electrocatalysts ultrasonically in a solution containing 2.49 mL of isopropyl alcohol (IPA, ≥ 99.7 %, Sigma-Aldrich), 2.49 mL of deionized water (D.I. water) and 20 μL of 5 wt.% Nafion ionomer (Nafion® 117 solution, Sigma-Aldrich). A sample loading of 10.2 $\mu g_{Ir} cm^{-2}$was achieved by depositing the appropriate amount of the suspension on the glassy carbon electrode and then drying in a convection oven at 50 $^{o}$C.

***The measurement of voltammetric charge and electrochemical porosity:*** According to Ardizzone and Trasatti, total charge $(q_T{}^*)$ and outer charge $(q_o{}^*)$ of metal oxide can be calculated by integration of voltammograms at different scan rates (2 – 300 $mV\ s^{-1}$).[1,2] Total charge $(q_T{}^*)$ corresponds with the whole active surface area of electrocatalyst, and can be obtained as the scan rate approaches to the zero. Outer charge $(q_o{}^*)$ shows the outer

accessible active surface during OER, and can be extrapolated as the scan rate approaches the infinite. The difference between $q_T^*$ and $q_o^*$ is the inner charge $(q_i^*)$ which is attributed to inner active surface area of oxide layer. Moreover, the ratio $q_i^*/q_T^*$ gives the information of electrochemical porosity which is related with the pore structure of electrocatalyst. To measure the voltammetric charge and electrochemical porosity, potential cycling was performed between 0.4 to 1.4 V vs. RHE at different scan rates (3000, 2000, 1000, 750, 500, 250, 100, 50, 20, 10 $mV\ s^{-1}$). The voltammetric charge corresponding to the total surface area $(q_T^*)$ can be calculated plotting the reciprocal of $q^*$ against the square root of the potential scan rate by using the following equation:

$$(q^*)^{-1}(v) = (q_T^*)^{-1} + kv^{1/2} \tag{E4}$$

The total voltammetric charge $(q_T^*)$ calculated by extrapolation of the linear plots at $\nu = 0$. The values of outer charge $(q_0^*)$ can be calculated from the extrapolation to $v \rightarrow \infty$ in the plot of $q^*$ versus $v^{-1/2}$ according to the following equation.

$$q^{-1}(v) = q_0^* + k'v^{-1/2} \tag{E5}$$

From $(q_T^*)$ and $(q_o^*)$ data, the inner voltammetric charge $(q_i^*)$ contribution of less accessible surface sites of the oxide layer to the total charge can be calculated.

$$q_T^* = q_i^* + q_0^* \tag{E6}$$

$$q_i^* = q_T^* - q_0^* \tag{E6'}$$

The electrochemical property defined as the relation between the inner and total charge as a function of the total charge.

$$\text{Electrochemical porosity} = {q_i^*}/{q_T^*} \quad \text{(E7)}$$

***Electrochemical stability test protocols:*** The stability test was performed by using the galvanostatic mode of 1 $mA\ cm^{-2}$ for 15 hour, under nitrogen condition. The rotating speed was 1600 rpm to remove the evaluated oxygen.

***4. MEA test:***

***Membrane electrode assembly fabrication:*** To prepare the membrane electrode assemblies (MEAs), a commercial 46% Pt/C catalyst purchased from TKK was used as the cathode, and the prepared catalysts were employed as the anode catalyst. The anode and cathode catalyst inks were prepared by ultrasonically blending with Nafion® solution (5% Sigma Aldrich) and isopropanol (IPA). This mixture was sprayed onto a NR-212 membrane (DuPant™, Nafion® PFSA Membrane) with 5 $cm^2$ of the geometric area. The total loading of Pt was fixed at 0.4 $mg_{Pt}\ cm^{-2}$ on the cathode and Ir loading was controlled at 1.0 $mg_{Ir}\ cm^{-2}$ on anode. After spraying the catalysts, the catalyst-coated membrane was hot pressed at 140 $^{o}$C for 2.5 min.

***Cell operation:*** The graphite bipolar-plate was used on cathode side and that of anode side was made from gold-coated titanium. The flow channel was designed to form serpentine type of flow field with a surface area of 5 $cm^2$. The depth and width of the cannel in the flow-field were both 1 mm. A gold-coated Ti-foam and carbon paper was used as current collectors

for anode and cathode respectively. The detail single cell structure was described in **Figure S7**. The prepared MEA was sandwiched between the current collectors and assembled in the cell body using 8 screws with a torque of 140 $kg_f\ cm$. PEMWE single cells were tested using a potentiostat (Bio-logic). The operation temperature of the cell was 80 $^oC$. The preheated de-ionized water at 80 $^oC$ supplied to both the anode and cathode compartments at a constant flow rate of 1.5 $mL\ min^{-1}$ without back pressure. The linear voltammetry profiles were performed for a range of potentials from 1.0 to 2.0 $V_{RHE}$ at a 5 $mV\ s^{-1}$ scan rate.

**References**


(1) De Pauli, C. P.; Trasatti, S. *Journal of Electroanalytical Chemistry* **1995**, *396*, 161.

(2) Hu, J.-M.; Zhang, J.-Q.; Cao, C.-N. *International Journal of Hydrogen Energy* **2004**, *29*, 791.

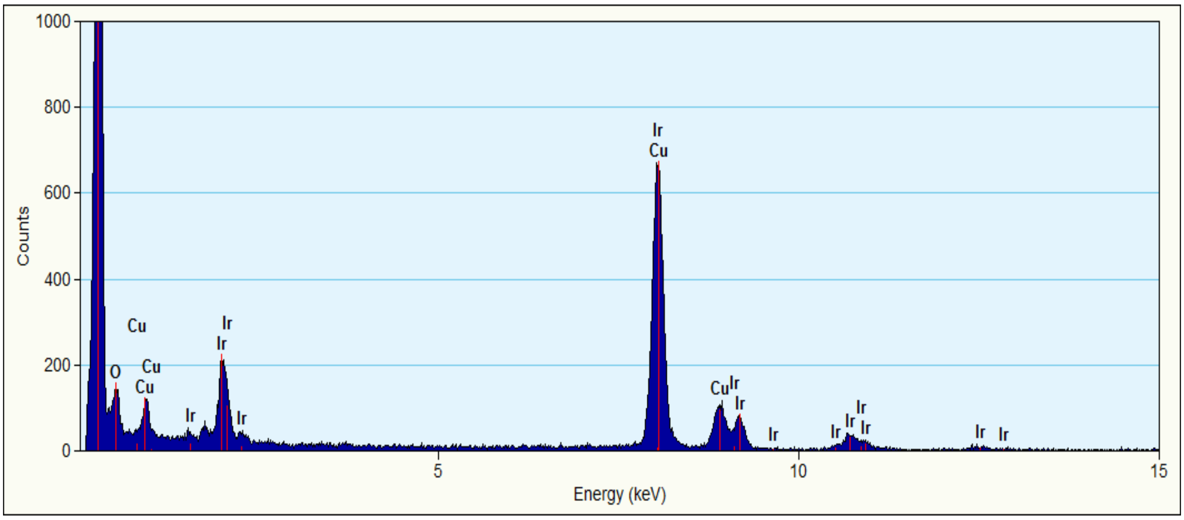


Figure S1. Energy dispersive X-ray spectroscopic (EDX) pattern of Ir nanodendrite (Ir-ND).

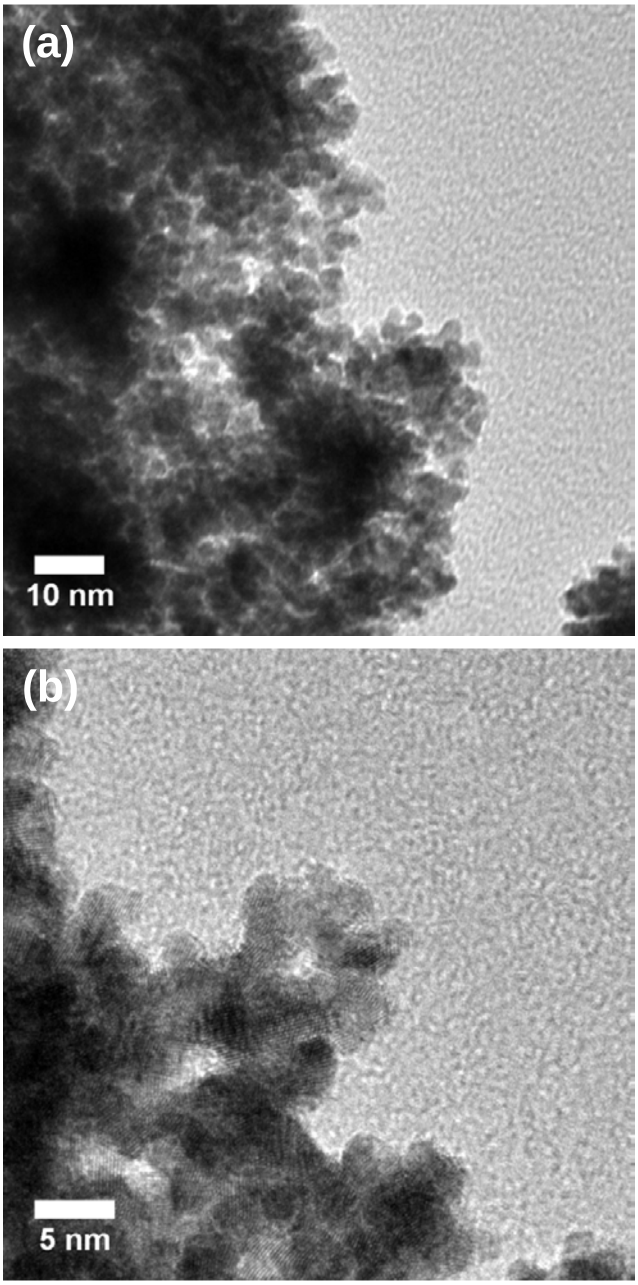


Figure S2. TEM images of commercial Ir black (Alfa Aesar, 99.8% metals basis) with different magnifications. (a) low and (b) high magnification.

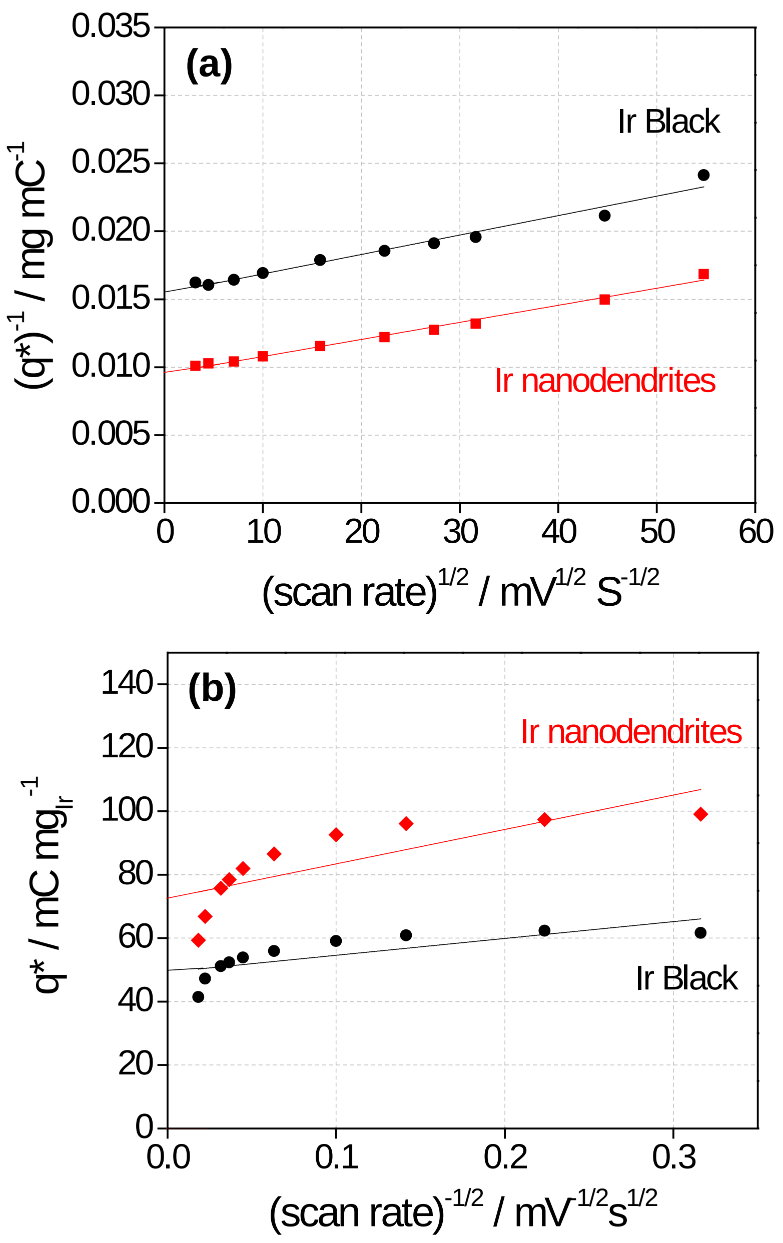


Figure S3. (a) Reciprocal voltammetric charge $(q^*)^{-1}$ versus the square root of the voltammetric scan rate $(\nu^{1/2})$. (b) Voltammetric charge $(q^*)$ versus the reciprocal square root of the voltammetric scan rate $(\nu^{-1/2})$ for Ir black and Ir nanodendrites (Ir-ND).

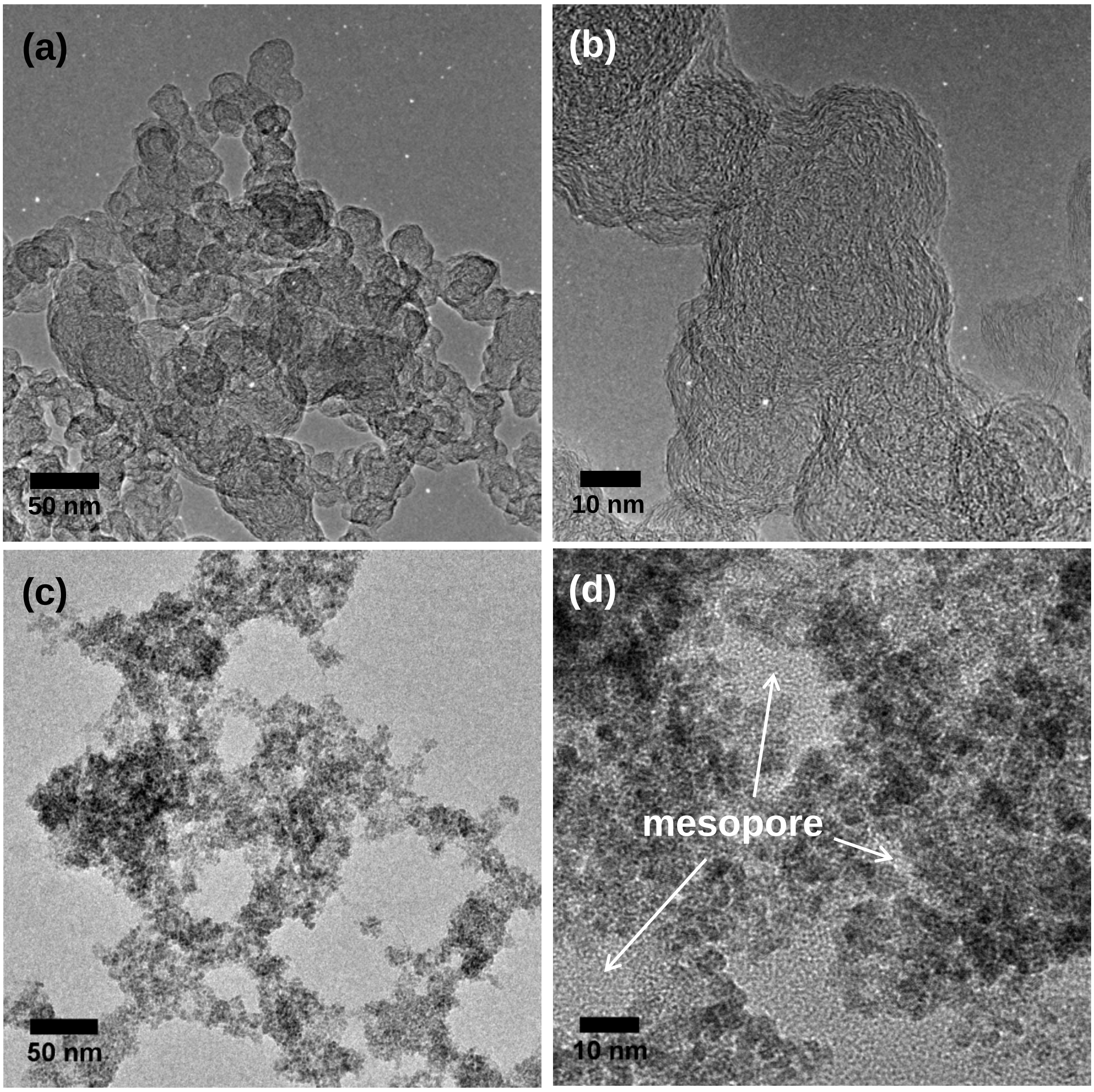


Figure S4. TEM images of bare catalyst supports. (a, b) carbon black (Vulcan® XC72R) and (c, d) synthesized antimony doped tin oxide (ATO).

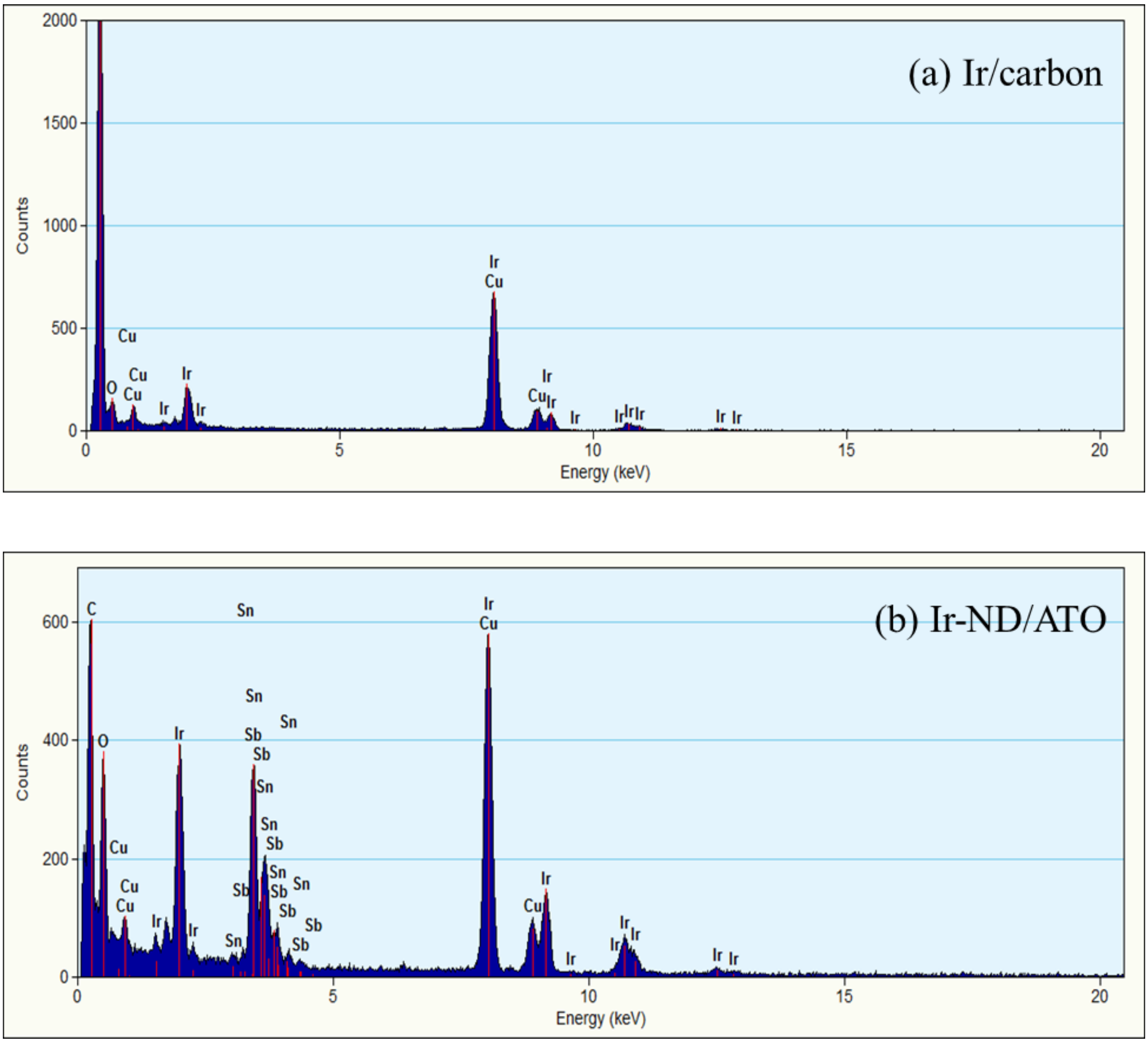


Figure S5. TEM based EDX patterns of (a) Ir/carbon and (b) Ir-ND/ATO.

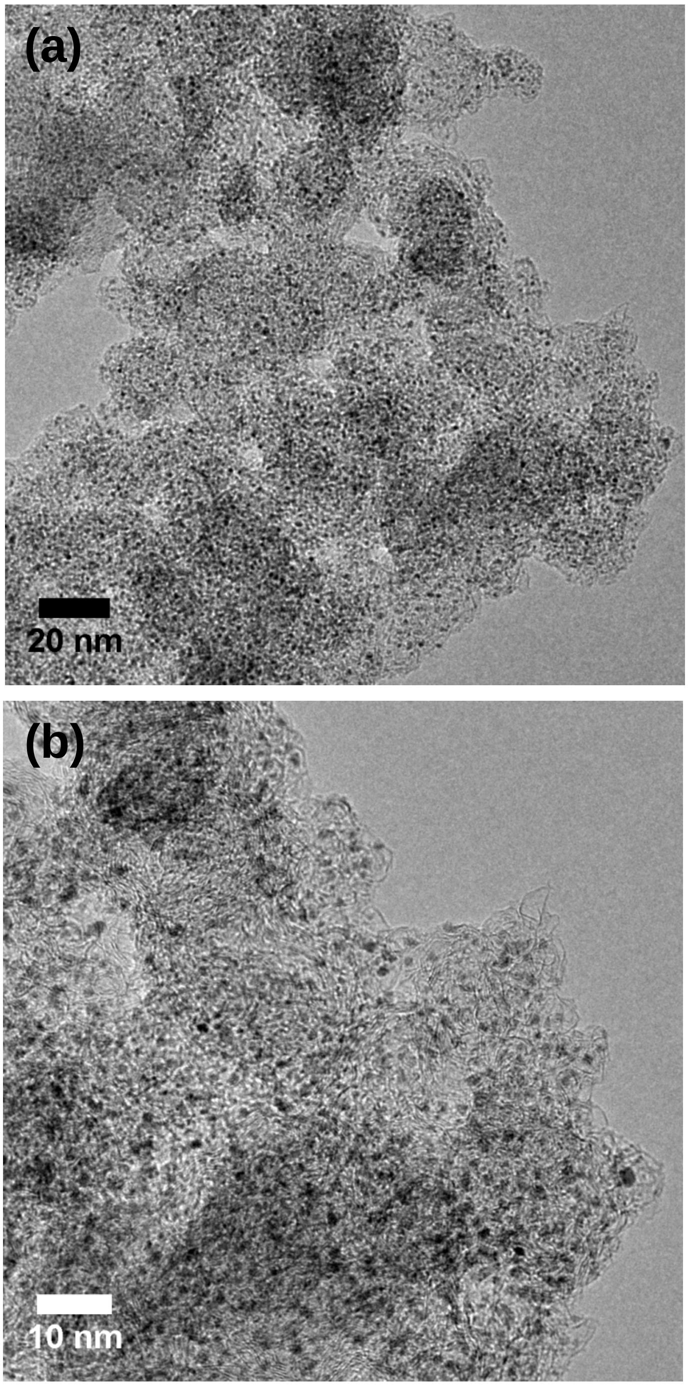


Figure S6. TEM images of synthesized Ir nanoparticles supported on carbon black with different magnifications. (a) low and (b) high magnification.

Table S1. Electrochemical properties of synthesized Ir-ND, Ir-ND/C and Ir-ND/ATO. For comparison, Ir black and Ir/C are also shown.

| Sample | Tafel slops (mV $dec^{-1}$) | Mass activity @ 1.51 $V_{RHE}$ (A $g_{Ir}^{-1}$) |
|---|---|---|
| Ir black | 56.8 | 8.0 |
| Ir/C | 55.6 | 31.1 |
| Ir-ND | 56.5 | 64.5 |
| Ir-ND/C | 57.7 | 67.9 |
| Ir-ND/ATO | 56.4 | 69.8 |

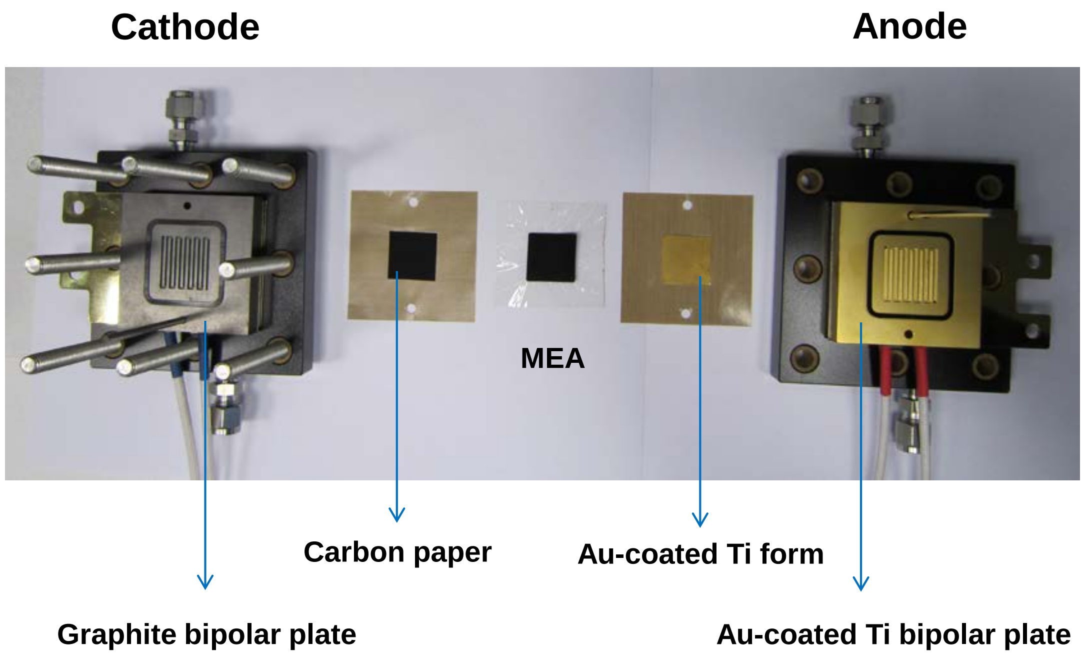


Figure S7. The picture of single cell for PEM water electrolysis. In the anode side, Au-coated Ti foam and plate were used as current collector and bipolar plate, respectively.
